# *ABCB4* is frequently epigenetically silenced in human cancers and inhibits tumor growth

**DOI:** 10.1038/srep06899

**Published:** 2014-11-04

**Authors:** Steffen Kiehl, Stefanie C. Herkt, Antje M. Richter, Liesa Fuhrmann, Nefertiti El-Nikhely, Werner Seeger, Rajkumar Savai, Reinhard H. Dammann

**Affiliations:** 1 Institute for Genetics; Justus-Liebig-University; Universities of Giessen and Marburg Lung Center, Member of the German Center for Lung Research; 35392 Giessen, Germany; 2 Molecular Mechanisms in Lung Cancer, Max Planck Institute for Heart and Lung Research, Member of the German Center for Lung Research; 61231 Bad Nauheim, Germany

## Abstract

Epigenetic silencing through promoter hypermethylation is an important hallmark for the inactivation of tumor-related genes in carcinogenesis. Here we identified the *ATP-binding cassette sub-family B member 4 (ABCB4)* as a novel epigenetically silenced target gene. We investigated the epigenetic regulation of *ABCB4* in 26 human lung, breast, skin, liver, head and neck cancer cells lines and in primary cancers by methylation and expression analysis. Hypermethylation of the *ABCB4* CpG island promoter occurred in 16 out of 26 (62%) human cancer cell lines. Aberrant methylation of *ABCB4* was also revealed in 39% of primary lung cancer and in 20% of head and neck cancer tissues. In 37% of primary lung cancer samples, ABCB4 expression was absent. For breast cancer a significant hypermethylation occurred in tumor tissues (41%) compared to matching normal samples (0%, p = 0.002). Silencing of *ABCB4* was reversed by 5-aza-2'-deoxycytidine and zebularine treatments leading to its reexpression in cancer cells. Overexpression of ABCB4 significantly suppressed colony formation and proliferation of lung cancer cells. Hypermethylation of *Abcb4* occurred also in murine cancer, but was not found in normal tissues. Our findings suggest that *ABCB4* is a frequently silenced gene in different cancers and it may act tumor suppressivly in lung cancer.

Epigenetic mechanisms play an important role for initiating and maintaining memory effects on gene expression. In mammalians, the epigenetic regulation is essential for normal development by regulating gene imprinting, X-chromosome inactivation and transcriptional inactivation of repetitive genomic elements. Moreover, epigenetic inactivation of tumor suppressor genes is frequently observed during carcinogenesis^1^. Especially hypermethylation of promoters harboring a CpG island is a hallmark of gene silencing during malignant transformation. CpG islands are sequences greater than 500 bp of GC-rich and CpG-dense elements in the genome. About 70% of known genes harbor a CpG island within the promoter and the first exon. During tumorigenesis CpG islands of the promoter regions of tumor suppressive genes become hypermethylated and this aberrant methylation is accompanied by the formation of repressive chromatin and gene inactivation. The most frequently epigenetically inactivated tumor suppressor genes are the *cyclin-dependent kinase inhibitor 2A* (p16) and the *Ras association domain family* (*RASSF*) genes^1^,^2^,^3^.

The ABC (ATP-binding cassette) transporters are a large family of transmembrane proteins, with seven subfamilies that are designated A to G^4^. These subfamilies are also termed ABC1, MDR/TAP, MRP, ALD, OABP, GCN20 and White, respectively. ABCB4 (ATP-binding cassette, sub-family B, member 4) belongs to the MDR/TAP subfamily and the protein is also known as MDR2 or MDR3 (multi drug resistance). ABC proteins transport various molecules (e.g. xenobiotics, drugs, lipid and other metabolic products) across the plasma and intracellular membranes^4^. ABCB4 is such a transporter and a member of the p-glycoprotein family of membrane proteins and translocates phospholipids (e.g. phosphatidylcholine) from the inner to the outer membrane of the hepatocyte^5^. The exact function of ABCB4 has not been determined in detail, however it is not involved in drug resistance of ovarian carcinoma cells to cisplatin^6^.

The *ABCB4* gene is localized on chromosome 7 at q21.12 (Fig. 1). Genetic alterations of *ABCB4* are associated with progressive familial intrahepatic cholestasis type 3, low phospholipid associated cholelithiasis and also found in women with intrahepatic cholestasis of pregnancy^7^,^8^,^9^,^10^,^11^,^12^. The ABCB4 protein consists of 1273 aa and harbors two ABC transporter transmembrane regions and two ATP-binding cassette domains (Fig. 1A). In accordance with its function, *ABCB4* is highly expressed in human liver, but lower mRNA levels were also found in other normal tissues^13^. The gene promoter of *ABCB4* harbors a CpG island that is usually unmethylated in normal cells (Fig. 1A)^14^. The epigenetic regulation *ABCB4* in cancer has not been analyzed in detail. In our study we report frequent hypermethylation of *ABCB4* in human cancers. Interestingly, we also observed a growth suppressive function of *ABCB4* in lung cancer cells.

## Results

### Methylation of *ABCB4* occurred in distinct human cancer entities

**Figure 1:**
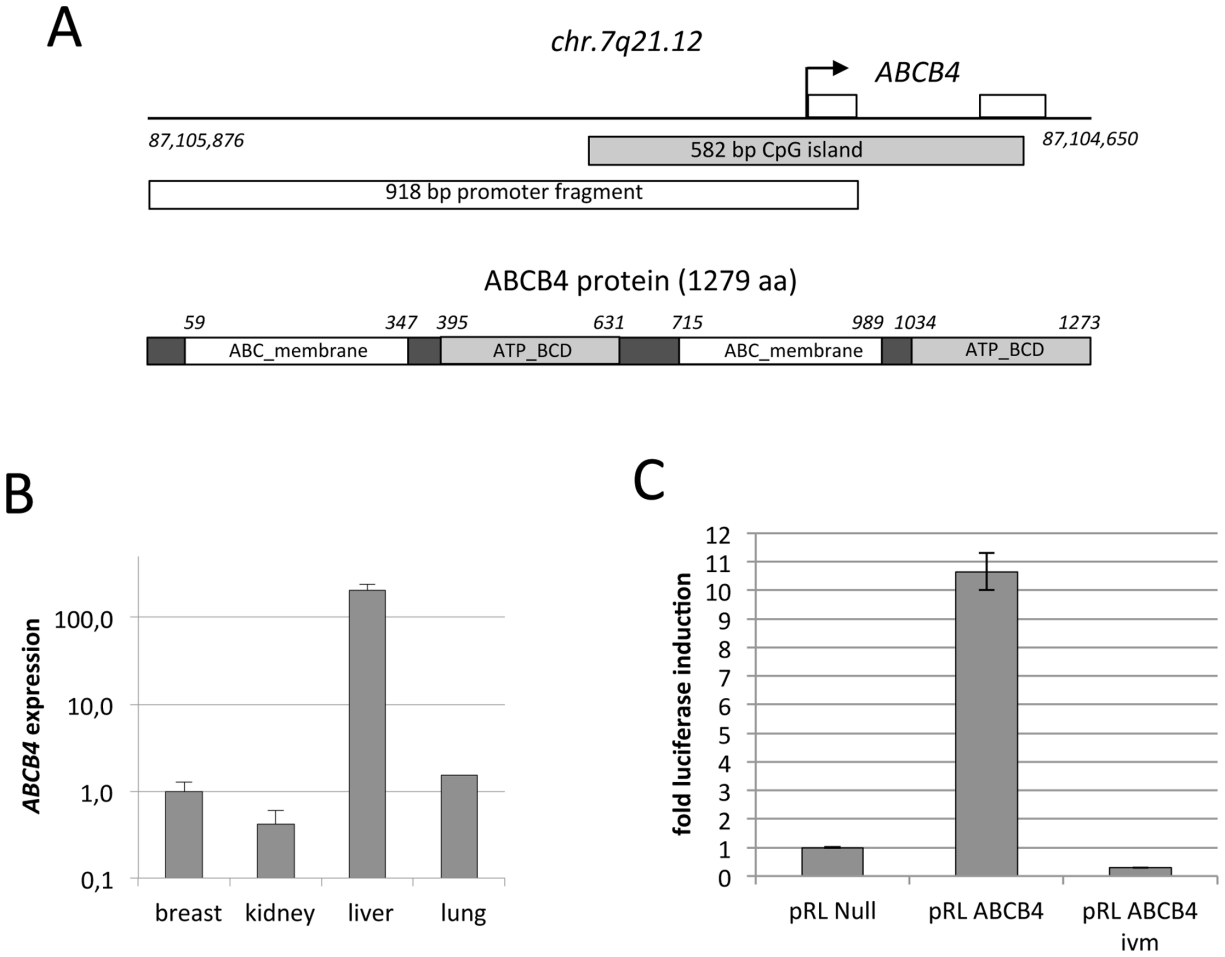
Epigenetic regulation of human *ABCB4*. (A). Structure of *ABCB4* CpG island promoter on chromosome 7q21.12 and the ACBC4 protein. Arrow marks transcriptional (+1) start site and boxes mark exons of ABCB4. The ABCB4 protein consists of two ABC transporter transmembrane regions (ABC_membrane) and two ATP-binding cassette domains (ATB_BCD). (B). *ABCB4* levels in normal tissues. Expression of *ABCB4* was analyzed in RNA isolated from normal lung, breast, liver and kidney samples by real-time RTPCR and normalized to *GAPDH* mRNA levels. (C). Promoter assay. HEK293 cells were transfected with 1 μg of pRL-null, pRL-ABCB4 and *in vitro* methylated (ivm) pRL-ABCB4 promoter construct and 0.35 μg of pGL3. Cells were isolated 24 h after transfection and studied using a dual-luciferase reporter assay.

We have performed a genome wide methylation screen (Infinium HumanMethylation450 BeadChip) in three lung cancer cell lines (A549, A427, H322) and normal human bronchial epithelial cells (NHBEC) and found a hypermethylation of *ABCB4* at six CpG sites in its CpG island promoter in A549, A427 and H322 compared to NHBEC (21%, 48% and 88% compared to 7%, respectively). Subsequently, we have analyzed the RNA levels of *ABCB4* in normal lung, breast, kidney and liver samples and detected expression of *ABCB4* in all four tissues (Fig. 1B). Expression of *ABCB4* in liver was found at much higher rate compared to the other three tissues. To analyze the impact of aberrant methylation at the *ABCB4* promoter, we have cloned a 918 bp promoter fragment into a luciferase reporter system and transfected it in HeLa and HEK293 cells. *In vitro* methylation of *ABCB4* drastically reduced the activity (36-fold reduction) of the promoter compared to the unmethylated promoter (Fig. 1C).

**Figure 2:**
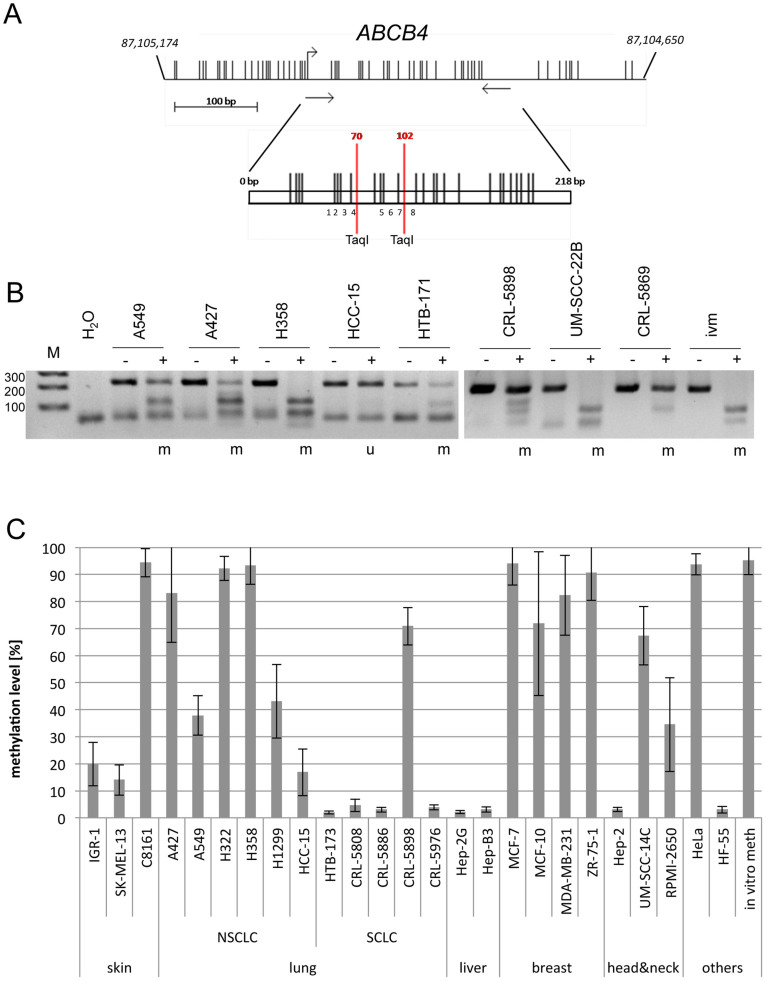
Methylation of *ABCB4* in human cancer cell lines. (A). CpG sites at the ABCB4 promoter regions are indicated as vertical lines. The PCR product (218 bp) with two *Taq*I sites (pos. 70 and 102) and eight CpG sites that were analyzed by pyrosequencing are depicted. (B). Combined bisulfite restriction analysis of *ABCB4*. Bisulfite-treated DNA from the indicated cancer cell lines and *in vitro* methylated DNA (ivm) were amplified, digested with *Taq*I (+) or mock digested (−) and resolved on 2% gels with a 100 bp marker (M); (m = methylated, u = unmethylated). (C). Bisulfite pyrosequencing of *ABCB4*. Methylation levels of eight CpGs of the bisulfite modified PCR products were analyzed by pyrosequencing.

To investigate the epigenetic status of *ABCB4* in human cancers in more details, we have analyzed its aberrant methylation in six non small cell lung cancers (A427, A549, H322, H358, HCC-15 and H1299), seven small cell lung cancers (HTB-173, CRL-5808, CRL-5976, CRL-5886, CRL-5898, CRL-5869, HTB-171), three breast cancers (MCF-7, ZR-75-1 and MDA-MB-231), skin cancer (IGR-1, SK-MEL-13, C8161), four head and neck (HN) cancers (Hep-2, UM-SCC-14C, UM-SCC-22B and RPMI-2650), two liver cancer (Hep-B3 and Hep-2G) cell lines, HeLa and human fibroblast (HF-55) by combined bisulfite restriction analysis (COBRA) and bisulfite pyrosequencing of eight CpG sites (Fig. 2). Fragmentation of the PCR product by *Taq*I indicates an underlying methylated *ABCB4* (Fig. 2B). *In vitro* methylated genomic DNA (ivm) served as a methylated control for COBRA and pyrosequencing (Fig. 2B and C). Normal human fibroblast (HF-55) and liver cancer cells (Hep-2G and Hep-B3) were unmethylated. For lung cancer, five (A427, A549, H322, H358 and H1299) out of six NSCLC cell lines were hypermethylated (>20% methylation) and three (HTB-171, CRL-5898 and CRL-5896) out of seven SCLC cell lines were methylated (Fig. 2B and 2C). Also three breast cancer cell lines (MCF-7, ZR-75-1 and MDA-MB-231), three HN cancers (UM-SCC-14C, UM-SCC-22B and RPMI-2650), skin cancer C8161 and HeLa exhibited *ABCB4* hypermethylation (>20%; Fig. 2B and C). Thus, 26 cancer cell lines were analyzed, of which 16 (62%) were methylated for *ABCB4* and its hypermethylation was found in different human cancer entities including lung, breast, skin and HN cancers.

### Tumor specific *ABCB4* hypermethylation in primary tumors and reduced ABCB4 expression in primary lung cancer

**Figure 3:**
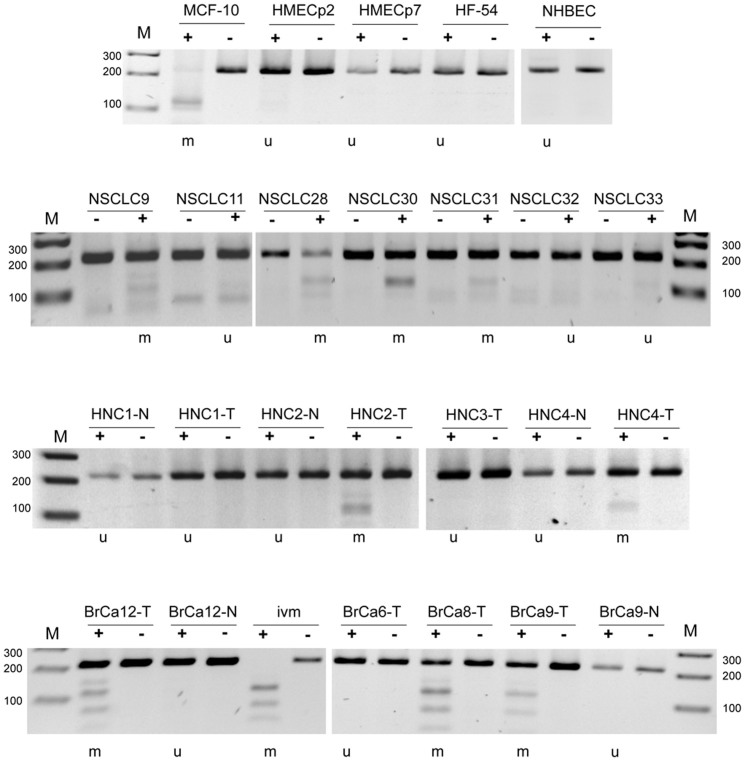
Methylation of *ABCB4* in primary tumors and normal tissues. Representative COBRA analysis is shown for normal human bronchial epithelial cells (NHBEC), human mammary epithelial cells (HMEC passage 2 and 7) and normal human fibroblast (HF-54) and primary non small cell lung cancer (NSCLC), head and neck cancer (HNC) and breast cancer (BrCa). Mock digest (−) and *Taq*I digest (+) are shown. Products were resolved on 2% gel with 100 bp marker (M). (T = tumor, N = corresponding normal tissue, ivm = in vitro methylated control, m = methylated, u = unmethylated).

Next we have analyzed the methylation levels of ABCB4 in normal human bronchial epithelial cells (NHBEC) and human mammary epithelial cells (HMEC) by COBRA (Fig. 3). HMEC, NHBEC and normal human fibroblast (HF-54) show no promoter methylation of *ABCB4* (Fig. 3). It is interesting to note that the MCF-10 cell line that served as a non-tumorigenic epithelial cell line also exhibited *ABCB4* hypermethylation (Fig. 2 and 3). However, MCF10 proliferates indefinitely in cell culture, since it was immortalized spontaneously^20^. To analyze the impact of epigenetic silencing of *ABCB4* in primary cancer tissues we investigated its hypermethylation in 46 primary NSCLC, 27 breast cancers and 10 HN cancers and several matching controls by COBRA. Representative results are shown in Figure 3. In HN cancer only two out of ten (20%) tumors (e.g. HNC2-T) exhibited *ABCB4* hypermethylation (Fig. 3). 18 out of 46 (39%) NSCLC (e.g. NSCLC30) showed partial methylation of *ABCB4* (Fig. 3). Moreover, 11 out of 27 (41%) breast cancer tissues (e.g. BrCa12-T) were methylated for *ABCB4* (Fig. 3). All 20 analyzed matching breast control tissue were unmethylated (e.g. BrCa12-N) (Fig. 3). For breast cancer a significant tumor specific methylation of *ABCB4* was found (p = 0.002, two tailed Fisher exact test).

Expression of ABCB4 was also analyzed on a lung cancer tissue array (data not shown). 44 out of 70 (63%) of primary lung cancers showed staining of ABCB4 and its expression was absent in 37% of cases. The highest expression of ABCB4 was observed in SCLC, while no staining was observed in mesotheliomas. ABCB4 staining was slightly higher in squamous cell carcinoma (18/30 or 60%) than in adenocarcinoma (8/14 or 57%). ABCB4 staining was dominantly cytoplasmic, but about 24% of the cases (mainly squamous cell cancer and SCLC) also showed nuclear staining. Neither absence of nuclear staining nor the cytoplasmic staining correlated with the tumor grade or clinical stage. In concordance with the methylation frequency (39%) of *ABCB4* in non small cell lung cancer, absence of its expression was observed in 41% (18/44) of primary NSCLC.

### Hypermethylation of *ABCB4 i*s associated with its downregulation in human cancer cell lines

**Figure 4:**
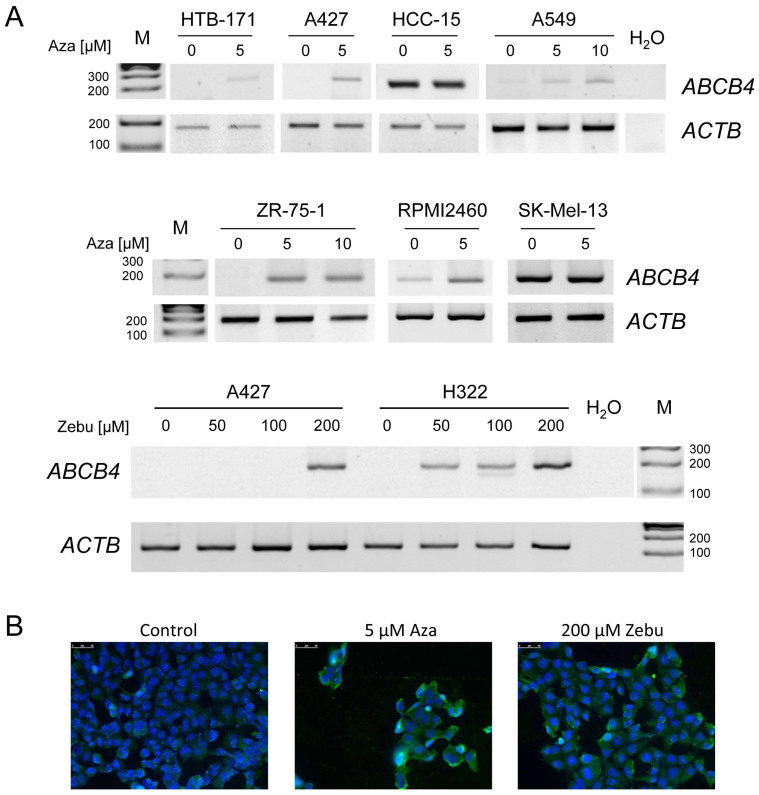
*ABCB4* expression in cancer cells, reexpression under DNMT inhibitor treatment. (A). Expression analysis of *ABCB4* is shown after 5-aza-2’-deoxycytidine (Aza) and zebularine (Zebu) treatments in lung cancer cell lines A427, A549, H322, HCC-15 and HTB-171, breast cancer ZR-75-1, head and neck cancer RPMI-2650 and melanoma SK-Mel-13. *ABCB4* (185 bp) and *ACTB* (226 bp) expression was analyzed by RT-PCR after four days of Aza or Zebu treatment on 2% gel with 100 bp marker (M). (B). Lung cancer H1299 cells treated with either 5 μM Aza or 200 μM Zebu for 72 h. Cells were blocked with 5% BSA for 1 h, followed by incubation with anti-ABCB4 antibody overnight at 4°C. Finally, cells were incubated with Alexa Fluor 488-conjugated secondary antibody and nuclei were stained with DAPI. Representative images are shown.

5-Aza-2'-deoxycytidine (Aza) and zebularine (Zebu) inhibit *de novo* methylation^21^,^22^ and are known to reverse hypermethylation of tumor suppressor genes, inducing their reexpression^2^. We therefore treated the lung cancer cell lines A427, A549, H322, HCC-15, HTB171 and H1299, the breast cancer cells ZR-75-1, the HN cancer cell RPMI-2650 and the melanoma cells SK-Mel-13 with Aza or Zebu and analyzed the *ABCB4* expression (Fig. 4). A427, A549, H322, HTB-171, ZR-75-1 and H1299 harbor a hypermethylated *ABCB4* promoter (>20% methylation Fig. 2) and show no endogenous *ABCB4* expression (Fig. 4). Treatment with 5 μM Aza or 200 μM Zebu lead to *ABCB4* reexpression on RNA level (Fig. 4A). Similar results were revealed for RPMI-2650, which are partially methylated (35% in Fig. 2) and *ABCB4* expression is increased upon Aza treatment (Fig. 4A). HCC-15 and SK-Mel-13 are unmethylated for *ABCB4* (<20% methylation; Fig. 2) and its expression is independent of the Aza treatment (Fig. 4A). Aza treatment of the lung cancer cells H1299 lead to ABCB4 reexpression on protein level (Figure 4B). In addition, reexpression of *ABCB4* in lung tumor cell lines A549, A427 and H322 was accompanied by *ABCB4* demethylation (data not shown). In cancer, we observed an epigenetic silencing of *ABCB4*, which was reversed by Aza or Zebu treatment.

### ABCB4 expression reduces colony formation and proliferation

**Figure 5:**
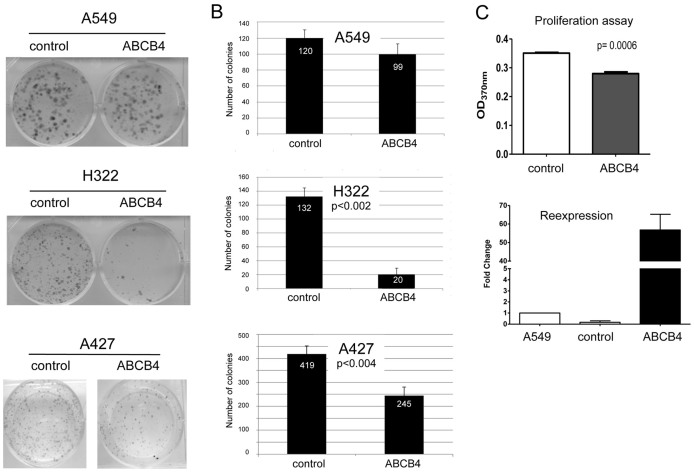
Overexpression of ABCB4 reduces colony formation and proliferation in lung cancer cell lines. (A). A549, A427 and H322 lung cancer cell lines were transfected with the ABCB4 expression construct or empty vector (pEGFP-C1), selected with G418 for 28 days and Giemsa stained. (B). Experiment was repeated three times and mean colony numbers with according SD are shown for A549, A427 and H322 cells. (C). Cell proliferation of A549 cells was evaluated by bromodeoxyuridine (BrdU) incorporation assay. BrdU incorporation was assessed by an ELISA reader. P values were calculated using two tailed paired t-test.

To functionally test ABCB4 and its ability to suppress tumor formation, we performed colony formation and proliferation assays (Fig. 5). Therefore, we transfected A427, A549 and H322 with an ABCB4 expression- or empty control construct (pEGFP) and selected with G418 for three weeks. Colonies that formed were Giemsa stained and representative pictures are shown (Fig. 5A). In all three cancer cell lines *ABCB4* is methylated (Fig. 2). Ectopic expression of ABCB4 in A549, H322 and A427 reduces the number of colonies (Fig. 5B). For H322 and A427 this growth suppressive effect was significant (p < 0.002 and p < 0.004, respectively; two tailed t-test). In addition, proliferation was also significantly reduced in A549 cells (p < 0.0006; Fig. 5C). These data suggest that ABCB4 exhibits a tumor suppressive function in human lung cancer cells.

### Epigenetic silencing of *Abcb4* in mouse cancer

**Figure 6:**
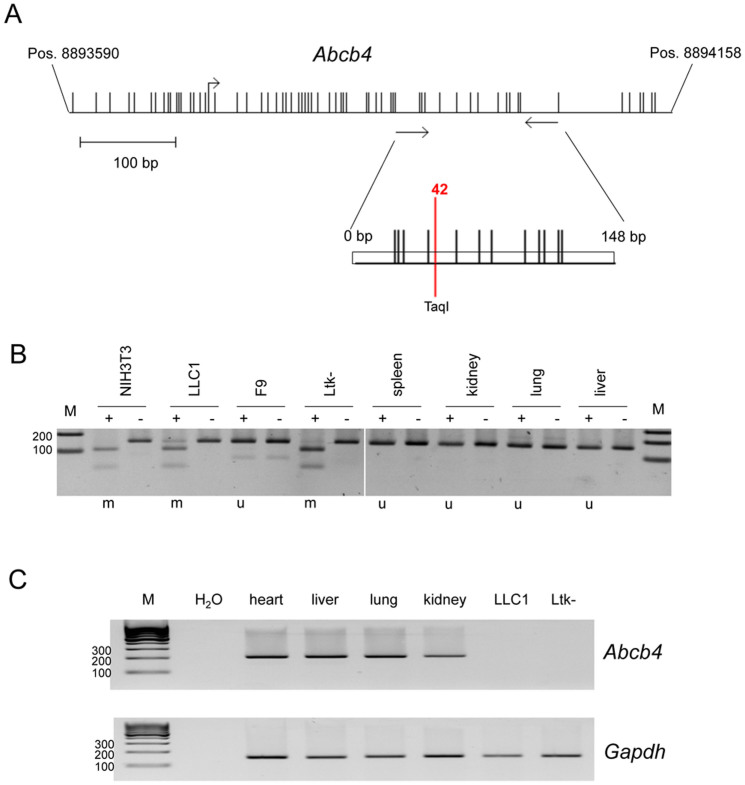
Epigenetic regulation of murine *Abcb4*. (A). Genomic organization of *Abcb4* CpG island on chromosome 5qA1. Transcriptional start, CpG sites and COBRA primers are depicted. The PCR product (148 bp) with the *Taq*I site (pos. 42) is indicated. (B). Methylation analysis of *Abcb4* by COBRA. Bisulfite converted DNA was amplified and PCR products were digested with *Taq*I (+) or mock-treated (−). Methylation status of *Abcb4* is indicated (m = methylated, u = unmethylated). (C). Expression analysis of murine *Abcb4* in heart, liver, lung and kidney and in murine cancer cell lines LLC1 and Ltk-. Expression of *Abcb4* (200 bp) and *Gapdh* (156 bp) was analyzed by RT-PCR on a 2% gel.

Subsequently, we have analyzed the methylation status and expression of the murine *Abcb4* gene (Fig. 6). *Abcb*4 is localized on chromosome 5qA1 and is transcribed from a 402 bp long CpG island promoter at position 8893614 to 8894015 (Fig. 6A). We have analyzed the methylation in normal mouse tissues (spleen, kidney, lung and liver) and in the murine cell lines: Lewis lung cancer 1 (LLC1), NIH3T3, teratocarcinoma F9 and Ltk- (Fig. 6B). Interestingly, in normal tissues *Abcb4* was unmethylated, but in three cell lines (LLC1, NIH3T3 and Ltk-) its hypermethylation was detected (Fig. 6B). NIH3T3 are primary mouse embryonic fibroblast cells, which were spontaneously immortalized and Ltk- was derived from the L929 murine fibrosarcoma cells. Moreover, we have analyzed the expression of *Abcb4* by RT-PCR (Fig. 6C). In normal tissues *Abcb4* expression was revealed, however in LLC1 and Ltk- expression of *Abcb4* was silenced. Thus, our data suggest that epigenetic silencing of *Abcb4* occurred also in pathogenesis of murine cancer.

## Discussion

In our study, we have identified the *ATP-binding cassette sub-family B member 4* (ABCB4) as novel epigenetically inactivated target gene in human cancer. Hypermethylation of *ABCB4* was found in several epithelial cancer entities including lung, breast, head and neck (HN), skin and cervix cancer. We also observed that aberrant methylation of *ABCB4* is also frequently found in primary NSCLC (39%), breast cancers (41%) and HN cancer (20%). In concordance with its methylation frequency absence of ABCB4 expression was observed in 41% primary NSCLC. To our knowledge hypermethylation of *ABCB4* was not reported in cancer previously. Methylation of *ABCB1* (MDR1) has been reported in breast cancer, HN cancer and other cancers^23^,^24^,^25^,^26^. Overexpression of several ABC transporter in cancer has been reported, however for ABCB4 and several other members (ABCA7, ABCA12, ABCB2, ABCB5 and ABCD1) downregulation in melanoma cell lines compared to normal melanocytes was revealed^27^. Thus, it will be interesting to analyze the methylation status of *ABCB4* in primary melanomas.

We have analyzed the expression of *ABCB4* in human tissues and observed the highest mRNA levels in liver. *ABCB4* expression was also found in normal breast, lung and kidney. This is in accordance with the previous observation that *ABCB4* is highly expressed in human liver, but ABCB4 expression was found in other normal samples (e.g. heart and muscle)^13^. In normal human bronchial epithelial cells, mammary epithelial cells and fibroblast *ABCB4* was unmethylated, but in primary tumor tissues aberrant promoter methylation of *ABCB4* was revealed (Fig. 3). In MCF10 cells that are utilized as a non-tumorigenic epithelial cell line, hypermethylation of *ABCB4* was found. Since MCF10 cells immortalized spontaneously and therefore proliferate indefinitely in cell culture^20^, MCF10 cannot be considered as normal human mammary epithelial cells. Normal breast epithelial cells undergo cellular senescence after few passages^20^,^28^,^29^. In MCF10 cells promoter hypermethylation of several tumor repressive genes, including *PTEN*, *p73*, *MGMT* and *cadherin* (*CDH1* and *CDH13*) has been reported^30^. Expression of mouse *Abcb4* was also observed in all normal tissues including heart, liver, lung and kidney (Fig. 6). In these tissues the *Abcb4* CpG island was unmethylated. We have also analyzed the methylation levels of *ABCB4*, in primary normal human liver tissues (data not shown). However, in these liver tissues and in the liver cancer cell lines Hep-B3 and Hep-2G no aberrant methylation of *ABCB4* was found (Fig. 2 and data not shown). Considering the ABCB4 transporter function of phospholipids from liver hepatocytes into bile, a high tissue specific expression of *ABCB4* in liver cells was expected and observed (Fig. 1). Mutations of *ABCB4* are associated with progressive familial intrahepatic cholestasis type 3, low phospholipid associated cholelithiasis and found in women with intrahepatic cholestasis of pregnancy^7^,^8^,^9^,^11^,^12^. However, some patients with *ABCB4* mutations also develop liver fibrosis, liver cirrhosis or cholangiocarcinoma^10^,^31^,^32^. Cholangiocarcinomas are malignant epithelial liver tumors with a poor prognosis and arise from the intra- and extra-hepatic bile ducts^33^. Since promoter hypermethylation of tumor associated genes has been reported in cholangiocarcinoma^33^,^34^, it could be important to investigate the methylation status of ABCB4 in this specific liver cancer entity. Abcb4 knock out mice develop chronic portal inflammation and bile duct proliferation with progression to liver fibrosis and hepatocellular carcinoma^35^. Other tumors in Abcb4 knock out mice have not been reported yet.

By using 5-aza-2'-deoxycytidine and zebularine treatments we were able to reexpress and demethylate *ABCB4* (Fig. 4 and data not shown). Moreover, methylation of the *ABCB4* promoter *in vitro* reduces its activity dramatically (Fig. 1). These results confirm that downregulation of *ABCB4* in cancer cells is due to its promoter hypermethylation. Aza (Decitabine) and its derivatives (e.g. Vidaza) are used for therapies of blood cancers^36^.

To verify the ability of ABCB4 to suppress tumor growth like, we performed colony formation and proliferation assays in lung cancer cell lines (Fig. 5). Our results show that ABCB4 significantly suppresses colony growth in H322 and A427 cancer cells, in which *ABCB4* is downregulated and inactivated by aberrant promoter methylation (Fig. 2 and Fig. 4). In A549 cells which show lower promoter hypermethylation levels compared to the other two cell lines growth suppression was less prominent, however proliferation was significantly reduced (Fig. 5C). Still, the exact function of ABCB4 in lung and breast epithelial cells has not been analyzed in detail. In ovarian cancer the resistance of ovarian carcinoma cells to cisplatin is not mediated by ABCB4^6^. Thus it will be interesting to further dissect the role of ABCB4 in the pathogenesis of cancer.

In summary we demonstrate that the *ATP-binding cassette sub-family B member 4* gene is frequently hypermethylated in human and murine cancers. Moreover aberrant methylation was frequently observed in primary lung and breast cancer samples. Demethylation of *ABCB4* is accompanied by its reexpression in cancer cell lines. Furthermore, ectopic expression of ABCB4 suppresses colony formation in lung cancer cells. Future research will elucidate the exact function of ABCB4 during carcinogenesis. It will be interesting to evaluate if aberrant *ABCB4* methylation may represent a novel biomarker for prognostic or diagnostic purposes in human cancer.

## Methods

### Tissue and cell lines

Primary cancer tissues and cancer cell lines were previously published^15^,^16^,^17^. All patients signed informed consent at initial clinical investigation. The study was approved by the local ethic committees (City of Hope Medical Center, Duarte, USA and Martin-Luther University, Halle, Germany). Mice tissues were obtained from C57/BL6 mouse strain. All cell lines were cultured in humidified atmosphere (37°C) with 5% CO_2_ and 1 × Penicillin/Streptomycin in according medium. Cells were transfected with 4 μg or 10 μg of constructs for 3.5 or 10 cm plates, respectively using Polyethylenimine (Sigma Aldrich) or Turbofect (Fermentas GmbH, St.Leon-Rot, Germany).

### Cell Proliferation assay

Cell proliferation was evaluated by BrdU incorporation using Cell Proliferation ELISA colorimetric kit (Roche Diagnostics) according to the manufacturer’s protocol. A549 cells were transfected with ABCB4-overexpression vector were seeded at a density of 10 × 10^3^/well in 96-well plates. After 24 h cells were starved in serum-free DMEM for further 24 h. BrdU was added to the cells and incubated for 4 h at 37°C. After removal of culture medium the cells were fixed and anti-BrdU antibody was added followed by the substrate. Finally BrdU incorporation was assessed by an ELISA reader.

### Methylation analysis

DNA was isolated by phenol-chloroform extraction and then bisulfite treated prior to COBRA analysis and pyrosequencing^18^. 200 ng were subsequently used for PCR with primer ABCB4BSU1 (GAGTAAAGTTTAGGTTTTTTTGTTGTAG) and 5’-biotinylated primer ABCB4BSL1Bio (CCTCAAAACCAAATACACCCTCTCC). Products were digested with 0.5 μl *Taq*I (Fermentas GmbH, St.Leon-Rot, Germany) 1 h at 65°C and resolved on 2% TBE gel. Methylation status was quantified utilizing the primer ABCB4BSseq (TTTAGAGGTTTTGTTAGATA) and PyroMark Q24 (Qiagen, Hilden, Germany). Eight CpGs are included in the analyzed region of *ABCB4* and mean methylation was calculated. For mouse Abcb4 the forward primer mABCB4BSU1 (GTGAAGTTTAGGTGAGGGAGGA) and the reverse primer mABCB4BSL1 (ACTACCTAAAAAAAAAAACCTCCAAA) were used and for *in vitro* methylation of genomic DNA M.SssI methylase was utilized (NEB, Frankfurt, Germany).

### Expression analysis

RNA was isolated using the Isol-RNA lysis procedure (5 Prime, Hamburg, Germany). 25 μg of breast, kidney, liver and lung RNA of normal human samples were obtained from Agilent Technologies (Waldbronn, Germany). RNA was DNase (Fermentas GmbH, St.Leon-Rot, Germany) digested and then reversely transcribed^19^. RT-PCR was performed with primers: ABCB4RTF1: GCAGACGGTGGCCCTGGTTGG, ABCB4RTR1: TGGAAAACAGCACCGGCTCCTG, 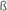
ACTFW: CCTTCCTTCCTGGGCATGGAGTC and 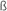
ACTRV: CGGAGTACTTGCGCTCAGGAGGA. For mouse mABCB4RTF1: CCCTCCAGCCGGCTTTCTCCA, mABCB4RTR1: GGACCGGAGCCTTGTGGTGAGG, mGAPDHRTF1: GCCGCCTGGAGAAACCTGCC and mGAPDHRTF1: CCCCGGCATCGAAGGTGGAA were utilized. Quantitative PCR (qRT-PCR) was performed in triplicates with PerfeCTa SYBR® Green (Quanta BioSciences, Gaithersburg, USA) using Rotor-Gene 3000 (Corbett Research, Qiagen, Hilden, Germany).

### Constructs

The cDNA of human ABCB4 was obtained as a full length cDNA vector IRATp970F09103D (Accession Number: BC042531; 4035 bp in pBluesriptR; imaGenes GmbH, Berlin, Germany) and the ORF of ABCB4 was cloned into the SacII and XmaI sites of pEGFP-C1. The *ABCB4* promoter was amplified from genomic DNA and cloned into pRLnull (Promega, Mannheim, Germany). Therefore a 918 bp promoter fragment was obtained by PCR with primers ABCB4Bgl2U1: GAGTCTTTTGGGAAGAGTGTGGAGGAATTA and ABCB4EcoRIL1: GAATTCGCGCGTGTCTGGCAGG and cloned into the BglII and EcoRI sites of pRLnull. *In vitro* methylation of the promoter construct was done with M.SssI methylase (NEB, Frankfurt, Germany).

### Promoter assay

HEK293 or HeLa cells were transfected with 1 μg of pRL-ABCB4 promoter construct and 0.35 μg of pGL3. Cells were isolated 24 h after transfection and studied using Dual-Luciferase Reporter Assay (Promega, Mannheim, Germany).

### Immunocytochemistry

Cells were seeded on chamber slides and treated with either 5 μM azacytidine or 200 μM zebularine for 72 h. After washing with PBS, cells were fixed with acetone-methanol (1:1) for 20 min at −20°C. Then cells were blocked with 5% BSA for 1 h, followed by incubation with anti-ABCB4 antibody (LifeSpan LS-B5729, 1:200) at 4°C overnight. Finally, cells were incubated with Alexa Fluor 488-conjugated secondary antibody, nuclei were stained with DAPI and mounted with fluorescent mounting media (Dako Cytomation, Glostrup, Denmark).

### Immunohistochemistry

Lung cancer tissue array were purchased from Pantomics, Inc. (Richmond, CA, USA). In brief, slides were treated with high pH antigen retrieval buffer. Rabbit anti-human ABCB4 (1:200; LS-B5729, lifespan biosciences, USA) and normal rabbit serum were used for stainings. Slides were incubated with ABCB4 antibody at 4°C overnight. After extensive washing, sections were incubate with secondary antibody, followed by staining with vector DAB (Vector Laboratories). The slides were washed and counterstained with hematoxylin for 5 min. All slides were analyzed under the Hamamatsu NDP slide scanner (Hamamatsu Nanozoomer 2.0HT) and its viewing platform (NDP. Viewer).

## Author Contributions

R.H.D., W.S. and R.S. have created the study. S.K., R.S. and R.H.D. participated in the design of the study. S.K., S.H., L.F., N.E.N. and A.M.R. acquired data. S.K., S.H., A.M.R., N.E.N., R.S. and R.H.D. controlled analyzed and interpreted data. R.H.D., R.S. and S.K. prepared the manuscript. S.K., S.H., A.M.R., R.S., L.F., N.E.N., W.S. and R.H.D. read, corrected and approved the final manuscript.

## Supplementary Material

Supplementary Information Supplementary Information
