# Supplementary material for: ABCB4 is frequently epigenetically silenced in human cancers and inhibits tumor growth
Source: Sci Rep. 2014 Nov 4;4:6899. doi: 10.1038/srep06899 (PMC4219162; doi:10.1038/srep06899)
Supplement: Supplementary Information [file srep06899-s1.pdf]

ABCB4 is frequently epigenetically silenced in human cancers and inhibits tumor growth

Steffen Kiehl[1], Stefanie C. Herkt[1], Antje M. Richter[1], Liesa Fuhrmann[2], Nefertiti El-Nikhely[2], Werner Seeger[2,3], Rajkumar Savai[2,3+] and Reinhard H. Dammann[1,3+]

[+]equal contribution

[1]Institute for Genetics; Justus-Liebig-University; Universities of Giessen and Marburg Lung Center, Member of the German Center for Lung Research; 35392 Giessen, Germany

[2]Molecular Mechanisms in Lung Cancer, Max Planck Institute for Heart and Lung Research, Member of the German Center for Lung Research; 61231 Bad Nauheim, Germany

[3]Member of the German Center for Lung Research (DZL)

Correspondence: Reinhard.Dammann@gen.bio.uni-giessen.de, Rajkumar.Savai@mpi-bn.mpg.de

Running title: ABCB4 silencing in human cancer

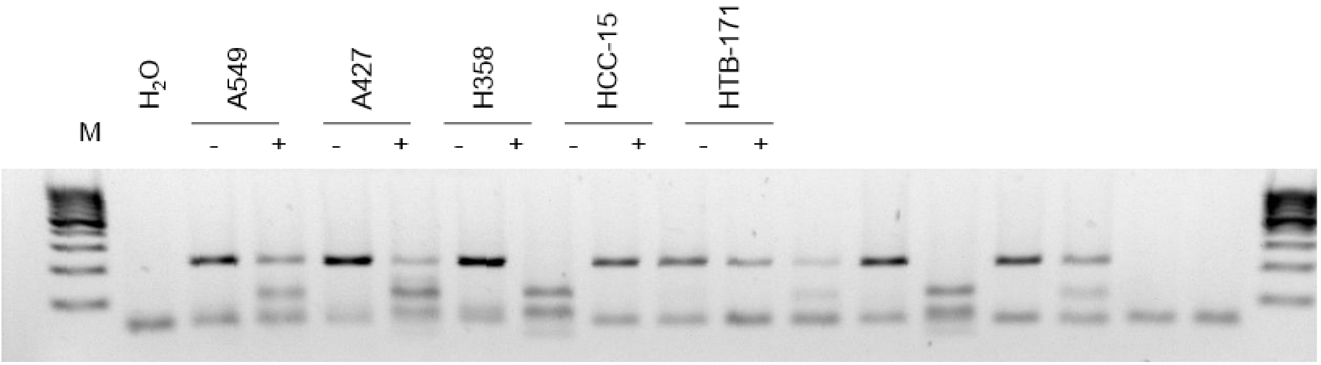

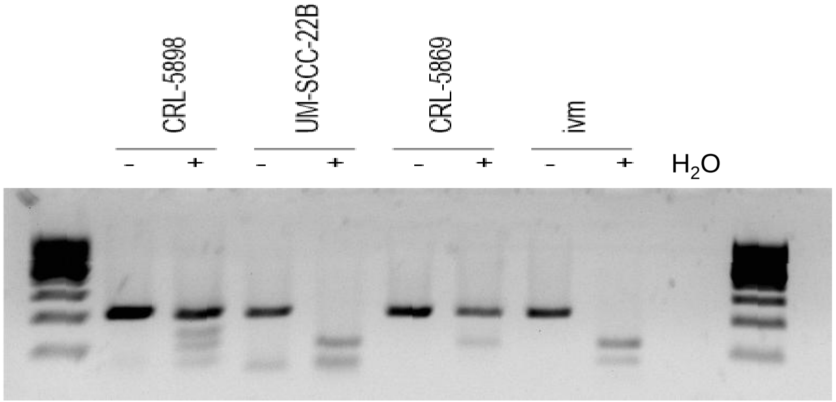

Supplement Figure 2

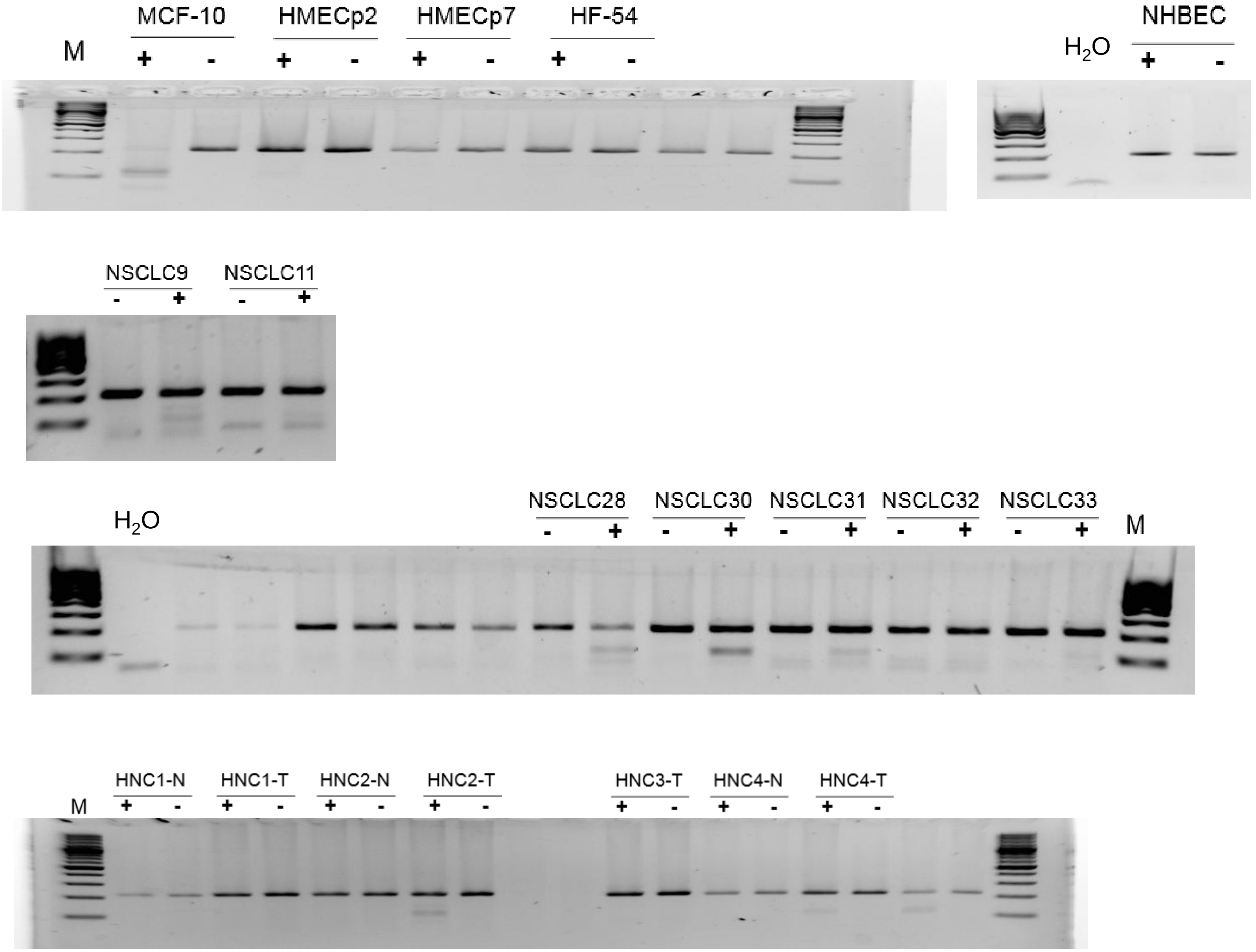

Supplement Figure 3

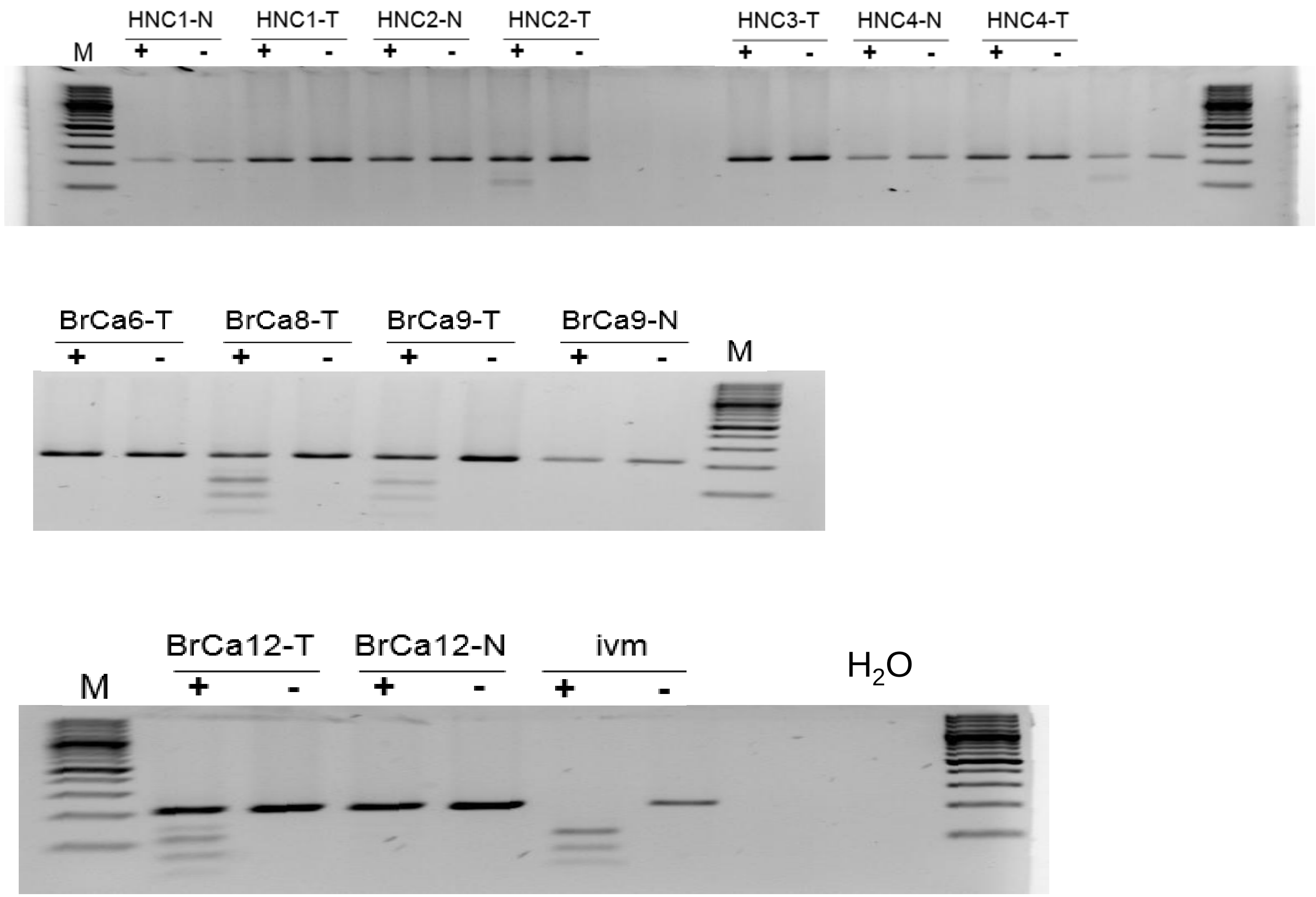

Supplement Figure 3

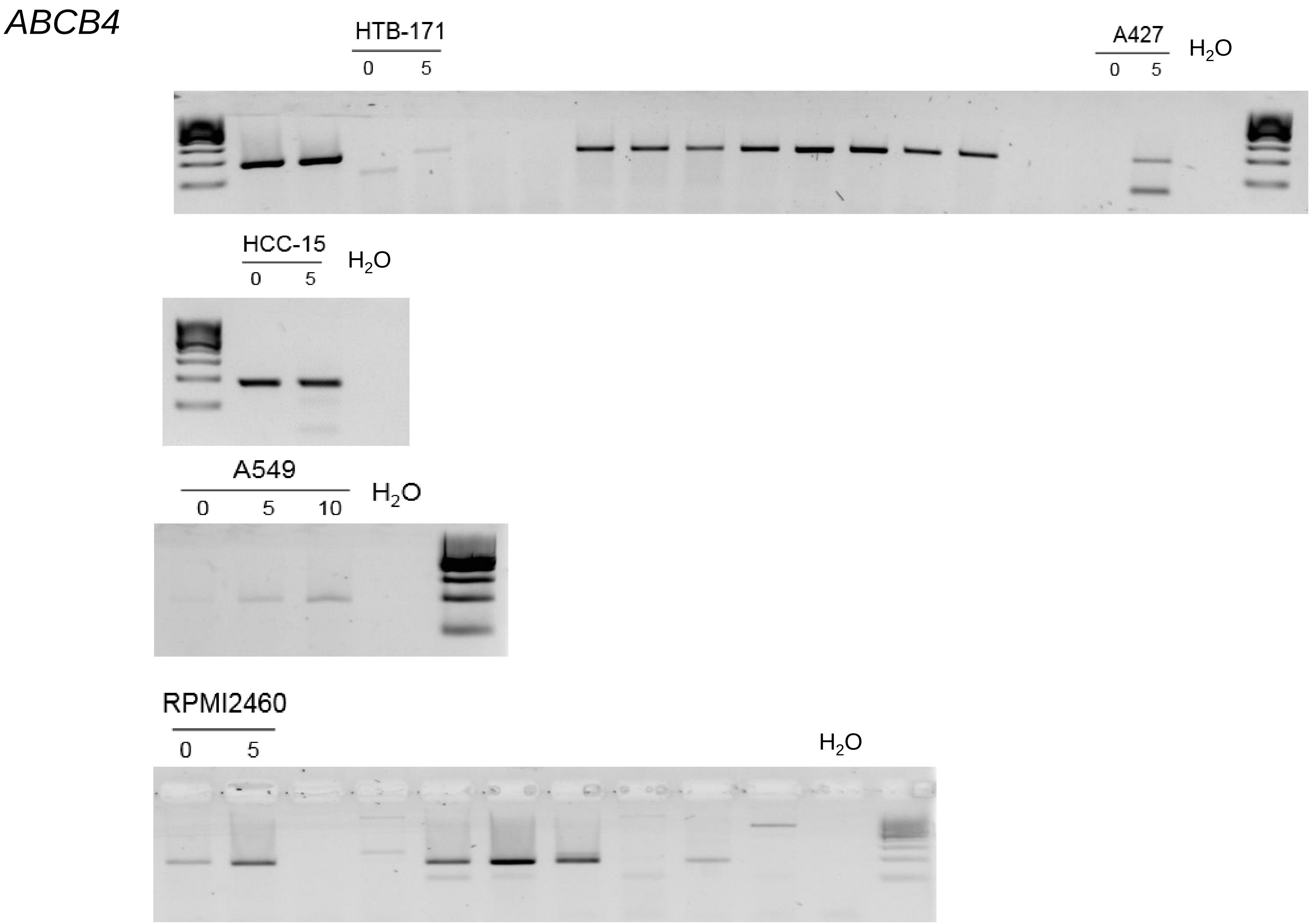

Supplement Figure 4

ABCB4

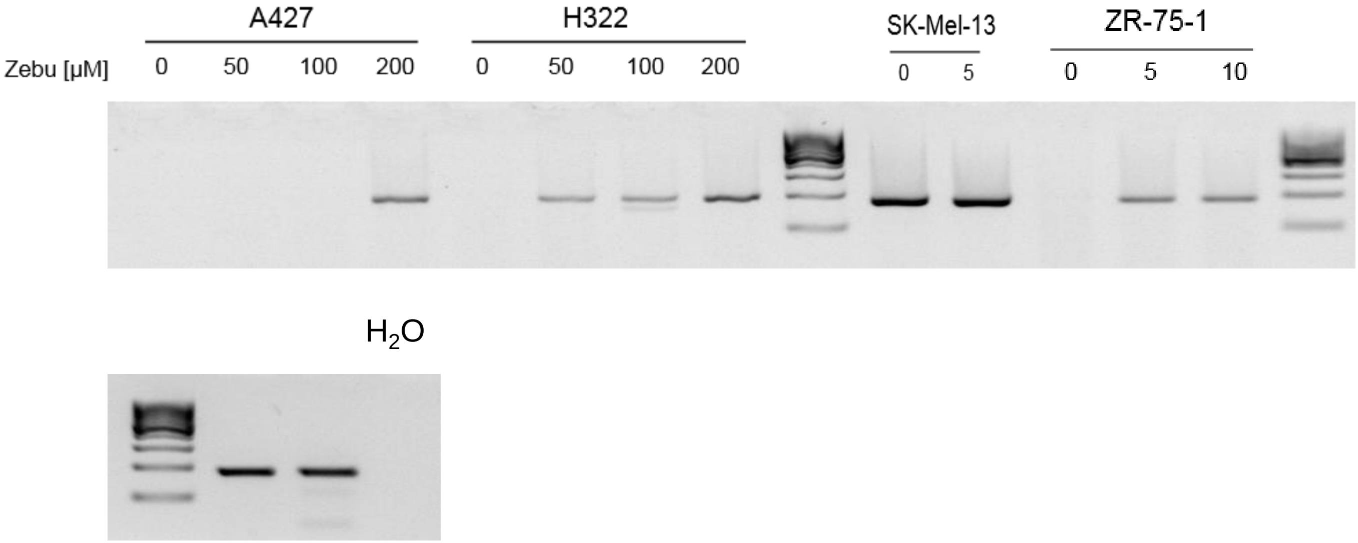

Supplement Figure 4

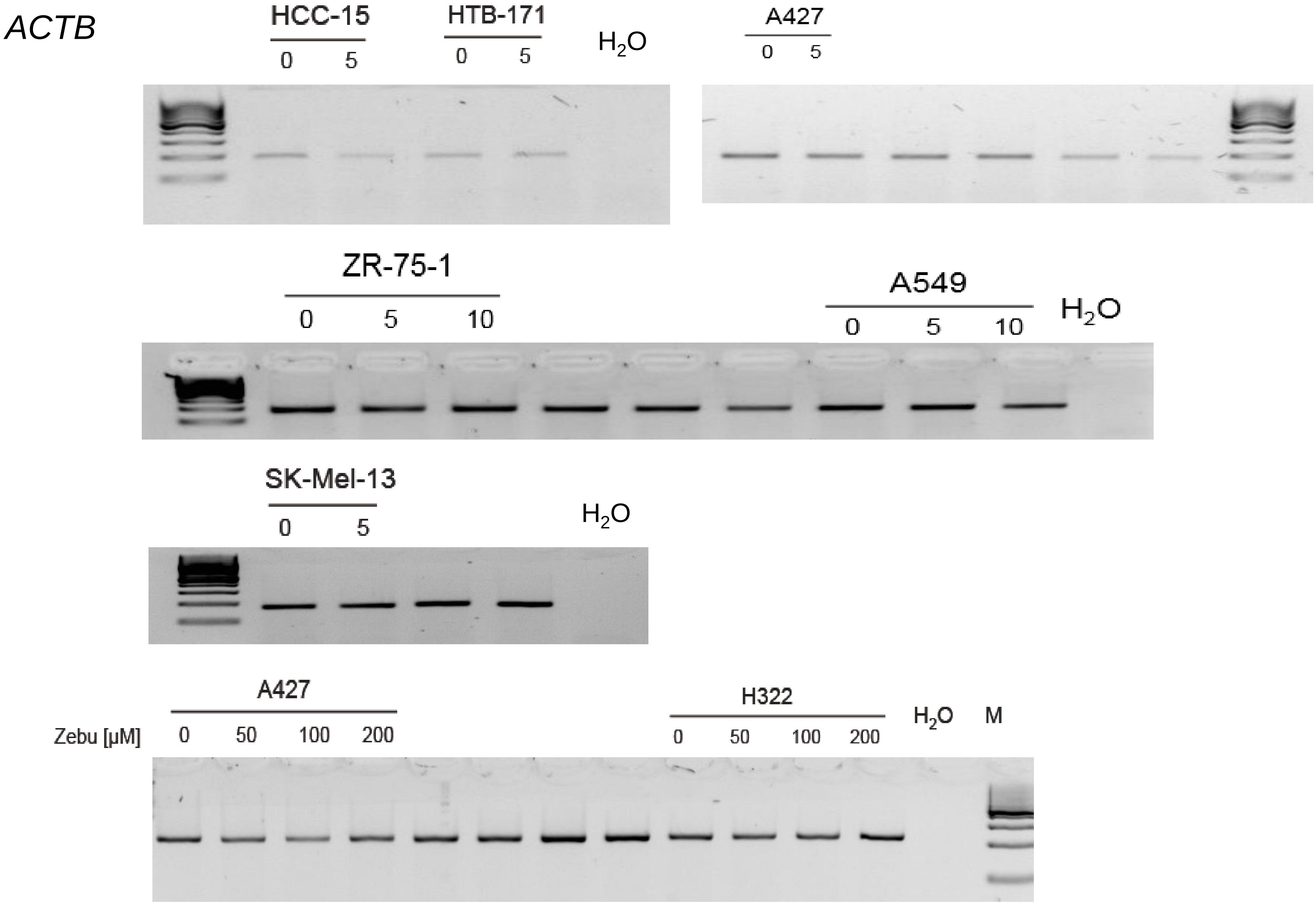

Supplement Figure 4

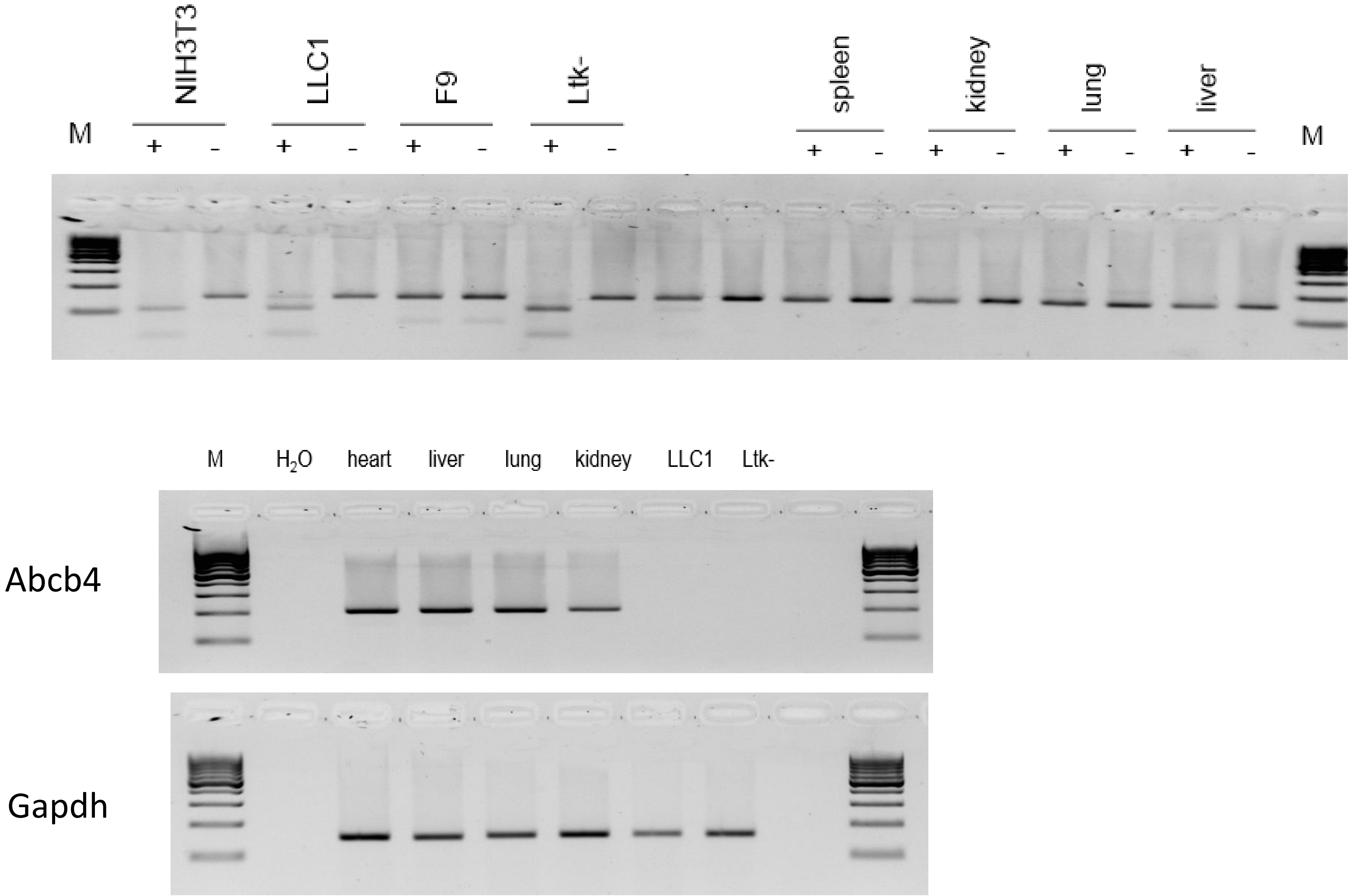

Supplement Figure 6
